# Postoperative hypotension in patients recovering from noncardiac surgery: a prospective, blinded observational study

**DOI:** 10.1016/j.bja.2026.04.042

**Published:** 2026-06-01

**Authors:** Moritz Flick, Simon Stemmler, Max Bossemeyer, Brian Überleer, Phillip Hoppe, Moritz Bünsch, Linda Krause, Kristen K. Thomsen, Bernd Saugel

**Affiliations:** 1 Department of Anesthesiology, Center of Anesthesiology and Intensive Care Medicine, University Medical Center Hamburg-Eppendorf, Hamburg, Germany; 2 Institute of Medical Biometry and Epidemiology, University Medical Center Hamburg-Eppendorf, Hamburg, Germany; 3 Outcomes Research Consortium®, Houston, TX, USA

**Keywords:** continuous monitoring, general ward, haemodynamic monitoring, postoperative complications, vital sign, wearable device

## Abstract

**Background:**

Postoperative complications can cause alterations in vital signs, including blood pressure. The duration and severity of postoperative hypotension, as well as its clinical relevance, remain poorly understood.

**Methods:**

In this single-centre prospective observational study, we quantified the incidence, duration, and severity of postoperative hypotension, defined as a mean arterial pressure (MAP) <65 mm Hg within the first 3 postoperative days, during recovery from noncardiac surgery. Postoperative blood pressure was monitored using blinded automated oscillometric blood pressure monitoring at 1-h intervals. On an exploratory basis, we also recorded postoperative complications within the first 7 postoperative days. Data are presented as median (25th, 75th percentile).

**Results:**

From February 2024 to July 2025, automated blood pressure monitoring was recorded in 1002 participants (age 58 [42–72] yrs; 50% female) for 48 (24, 64) h. Postoperative hypotension was recorded in 378/1002 (37.7%) participants, with a median cumulative duration of 180 (71, 420) min, corresponding to 7.1% (3.3%, 16.3%) of their monitoring time. The median area under a MAP of 65 mm Hg was 836 (300, 2037) mm Hg x min. The median time-weighted average MAP <65 mm Hg was 0.32 (0.12, 0.73) mm Hg. The incidence of postoperative hypotension progressively decreased from 30% of participants on postoperative day 1 to 17% of participants on postoperative day 3. Participants with postoperative hypotension had higher rates of acute kidney injury and unplanned admissions to the intensive care unit.

**Conclusions:**

During recovery from noncardiac surgery, postoperative hypotension is common, but rarely severe or prolonged. The incidence of postoperative hypotension progressively decreases from postoperative day 1 to day 3.

**Editor’s key points**

• Little is known about postoperative hypotension beyond the critical care environment.
• This single-centre prospective observational study quantified the incidence, duration, and severity of hypotension (MAP <65 mm Hg) within the first 3 days after noncardiac surgery.
• Postoperative blood pressure was recorded by automated oscillometric blood pressure monitors every hour.
• Postoperative hypotension was recorded in 378/1002 (37.7%) participants, and still occurred in 17% on postoperative day 3.
• Although common, postoperative hypotension was typically not severe or prolonged.

Around 2% of patients having major inpatient surgery die within the first 30 postoperative days, with most residing in general wards during their initial hospital stay.^1^, ^2^, ^3^, ^4^ Postoperative deaths are frequently a direct or indirect consequence of complications such as major bleeding, cardiovascular events, or sepsis.^3^^,^^5^, ^6^, ^7^, ^8^ All these major complications can result in lower blood pressure.

In patients recovering from surgery on wards beyond the monitoring environment afforded by critical care, blood pressure is typically measured much less frequently (every 4–8 h).^9^ Consequently, the duration and severity of postoperative hypotension—as well as its clinical relevance—remain poorly understood.^6^^,^^10^^,^^11^

In this single-centre, prospective observational study, we aimed to quantify the incidence, duration, and severity of postoperative hypotension in patients recovering from noncardiac surgery using blinded automated oscillometric blood pressure monitoring at 1-h intervals. On an exploratory basis, we also assessed the relationship between postoperative hypotension and complications, including acute kidney injury and unplanned intensive care unit admission.

## Methods

### Study design

This single-centre prospective observational study was conducted between February 2024 and July 2025 at the Department of Anesthesiology, Center of Anesthesiology and Intensive Care Medicine, University Medical Center Hamburg-Eppendorf, Hamburg, Germany. The study was approved by the ethics committee (Ethikkommission der Ärztekammer Hamburg, Hamburg, Germany) on June 7, 2021 (registration number: 2021–10423-BO-ff). Written informed consent was provided by all participants. We report the study according to the STROBE (STrengthening the Reporting of OBservational studies in Epidemiology) guideline.^12^

### Inclusion criteria

We enrolled adults (≥18 yr old) scheduled for elective noncardiac surgery (expected to last ≥90 min) with general anaesthesia.

### Exclusion criteria

We excluded people with planned postoperative admission to the intensive care unit, pregnant women, and people with contraindications for automated upper-arm cuff oscillometry.

### Blinded automated postoperative blood pressure monitoring

We measured blood pressure at 1-h intervals up to 3 postoperative days using automated upper-arm cuff oscillometry. In participants who were transferred to the general ward after a short stay in the standard post-anaesthesia care unit (PACU), blood pressure was measured using an OnTrak device (Spacelabs Healthcare, Snoqualmie, WA, USA) throughout the entire study period. In participants who were first admitted to an advanced PACU for overnight monitoring, blood pressure was measured hourly with the patient monitor (IntelliVue X3; Philips, Amsterdam, The Netherlands) in the advanced PACU and then with an OnTrak device after transfer to the general ward. In the advanced PACU, a team of anaesthesiologists and nurses provides high-acuity monitoring and care for surgical patients during the remaining day of surgery and the first postoperative night to make patients fit for transfer to a general ward by the morning of the first postoperative day.

Clinicians and participants were blinded to automated oscillometric blood pressure measurements on the general ward. Blood pressure measurements, therefore, did not influence clinical care. Blood pressure data were exported from the OnTrak device using the Sentinel software (Spacelabs Healthcare). We excluded artefactual measurements – defined as values flagged as ‘erroneous’ by the OnTrak device or those falling outside the predefined physiologic ranges: diastolic blood pressure <30 mm Hg or >200 mm Hg and systolic blood pressure <60 mm Hg or >260 mm Hg. A measurement was considered valid until the next measurement—typically performed after a 1-h interval. The final measurement of each patient was considered valid for 1 h.

### Endpoints

We defined ‘postoperative hypotension’ as a mean arterial pressure (MAP) <65 mm Hg. We quantified postoperative hypotension using the following endpoints: the proportion of participants with at least one MAP <65 mm Hg (primary endpoint); the cumulative duration participants had MAP <65 mm Hg; the percentage of monitoring time with MAP <65 mm Hg; the area under a MAP of 65 mm Hg; and the time-weighted average MAP <65 mm Hg.

To further quantify blood pressure within the first 3 postoperative days, we additionally assessed the following endpoints: All endpoints were additionally assessed for MAP thresholds of <75 mm Hg and <55 mm Hg, and evaluated both cumulatively over the first 3 postoperative days and separately for the remainder of the day of surgery and postoperative days 1, 2, and 3.

### Exploratory outcomes

On an exploratory basis, we describe how many participants with and without postoperative hypotension (defined as a MAP <65 mm Hg) within the first 3 postoperative days had postoperative complications within the first 7 postoperative days or until hospital discharge (whatever occurred first). Specifically, we analysed complications according to Clavien-Dindo,^13^ acute kidney injury (defined as a serum creatinine increase of at least 0.3 mg dl^-1^ within 48 h or from the last preoperative value or serum creatinine increase of 1.5 times from the last preoperative value within 7 days),^14^ and unplanned intensive care unit admission. We also assessed hospital length of stay and 30-day mortality. Data on complications were extracted from the participants’ electronic medical records. Thirty-day mortality was assessed via follow-up telephone interviews.

### Statistical analysis

We used R version 4.4.3 (R Foundation for Statistical Computing, Vienna, Austria) for statistical analyses. We used descriptive analyses to quantify participant characteristics, procedural data, and postoperative blood pressure. Categorical data are presented as numbers (%), and continuous data as median (25^th^–75^th^ percentile).

The monitoring duration was defined as the time between the first and the last postoperative blood pressure measurement. As the primary endpoint, we analysed the number of participants with any MAP measurement below the threshold. Further, we analysed the absolute cumulative duration below threshold (by summing up all episodes below threshold), the relative duration below threshold (by dividing the cumulative duration below threshold by the total monitoring time), the area under threshold, and the time-weighted average MAP. We calculated areas under a threshold by subtracting the measured value from the predefined threshold and summing all the positive values (mm Hg x min). Time-weighted average MAP below thresholds was calculated as area under a MAP threshold divided by the monitoring time. Additional analyses were performed separately for the remaining day of surgery, postoperative day 1, postoperative day 2, and postoperative day 3.

Comparisons between participants with and without postoperative hypotension (defined as a MAP <65 mm Hg) were analysed using Pearson’s χ^2^ test with Yates’ continuity correction or Kruskal-Wallis rank sum test as appropriate. *P*-values are used as descriptive summary measures, are not adjusted for multiple testing, and thus should not be interpreted as results of confirmatory hypothesis testing.

We did not perform a formal sample size calculation due to the limited data on postoperative hypotension at the time of planning this study. Based on an estimated incidence of about 20–25% of postoperative hypotension in this noncardiac surgery patient cohort, we assumed that a sample size of 1000 participants would be sufficient to address our study objective. Due to unplanned changes, postponements or cancellations in the surgical schedule, we expected a dropout rate of about 20% and thus planned to include 1300 participants.

## Results

### Study characteristics

**Fig 1:**
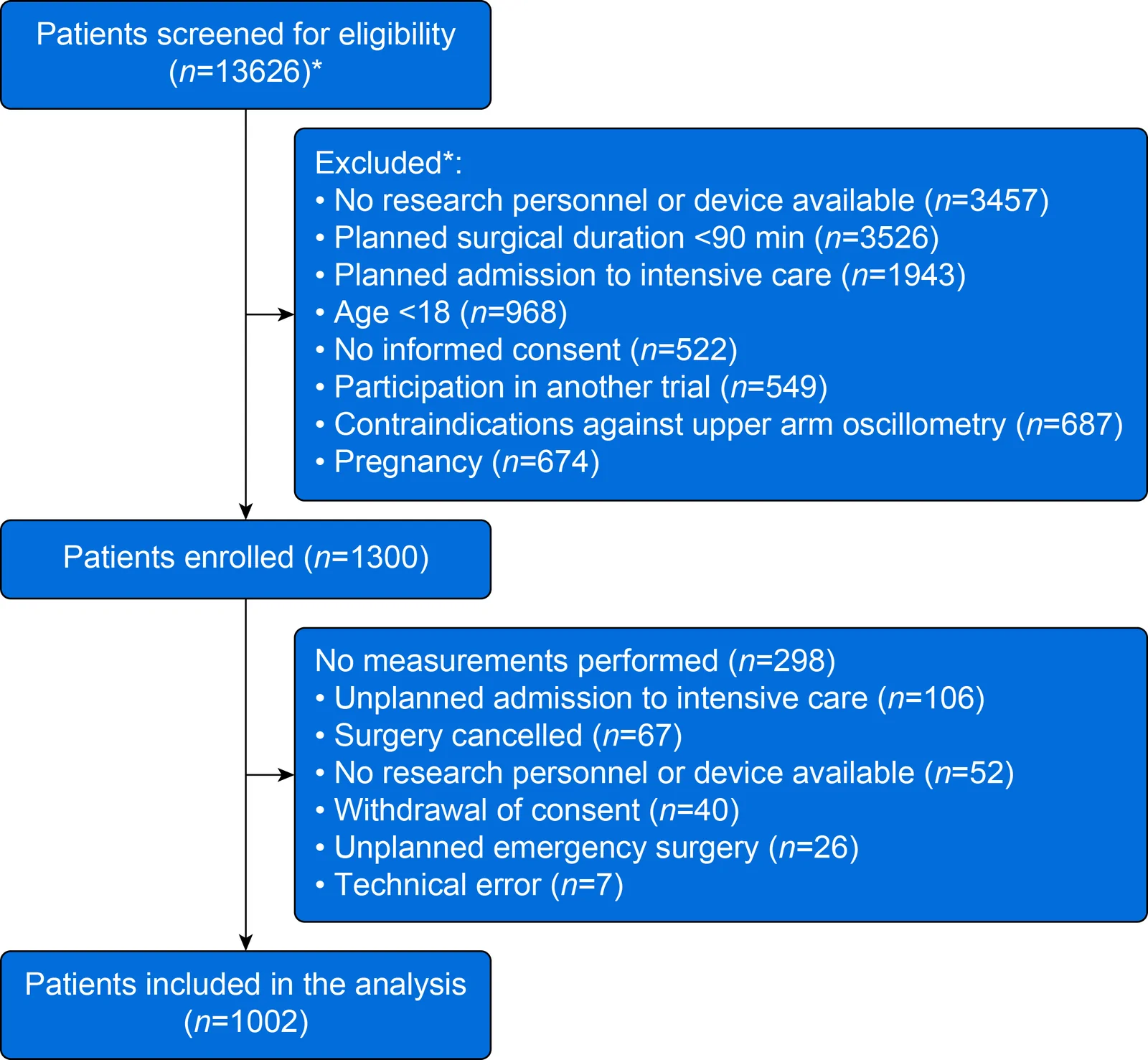
Study flowchart. **∗**Numbers were extrapolated from 1 month to the full study period.

As planned, we enrolled 1300 participants, with 1002 participants included in the final analysis (Fig. 1, Table 1). The median (25^th^, 75^th^ percentile) monitoring duration was 48 (24, 64) h (2859 [1410, 3838] min). Missing blood pressure measurements occurred in 862/1002 (86.0%) participants, with a median number of missing values per participant of 3 (1, 7).

**Table 1:** Participant characteristics and procedural data. Data are shown as absolute number (percentage) or median (25th, 75th percentile).

		With postoperative hypotension (*n*=378)	Without postoperative hypotension (*n*=624)
*Participant characteristics* Male sex, *n*		173 (46%)	327 (52%)
Age, yr		60 (43, 72)	57 (42, 67)
Body mass index, kg m^−2^		26 (23, 30)	26 (23, 30)
ASA physical status, *n*	1	30 (8%)	63 (10%)
	2	196 (52%)	348 (56%)
	3	142 (38%)	206 (33%)
	4	10 (3%)	7 (1%)
Revised cardiac risk index, *n*	0	277 (73%)	518 (83%)
	1	60 (16%)	84 (13%)
	2	29 (8%)	17 (3%)
	3+	12 (3%)	5 (1%)
Modified 5-Item frailty index, *n*	0	214 (57%)	373 (60%)
	1	110 (29%)	188 (30%)
	2	42 (11%)	49 (8%)
	3	7 (2%)	13 (2%)
	4	4 (1%)	1 (0%)
	5	1 (0%)	0 (0%)
Arterial hypertension, *n*		141 (37%)	222 (36%)
Coronary artery disease, *n*		47 (12%)	28 (4%)
Chronic heart failure, *n*		26 (7%)	12 (2%)
Chronic obstructive pulmonary disease, *n*		12 (3%)	16 (3%)
Diabetes mellitus, *n*		41 (11%)	56 (9%)
Atrial fibrillation, *n*		26 (7%)	32 (5%)
*Type of surgery*			
General surgery, *n*		98 (26%)	148 (24%)
Neurosurgery, *n*		63 (17%)	127 (20%)
Trauma surgery, *n*		67 (18%)	93 (15%)
Urology, *n*		51 (13%)	91 (15%)
Otorhinolaryngology, *n*		34 (9%)	58 (9%)
Gynaecology, *n*		34 (9%)	48 (8%)
Oral and maxillofacial surgery, *n*		21 (6%)	46 (7%)
Other, *n*		10 (3%)	13 (2%)
Duration of surgery, min		121 (74, 181)	116 (75, 173)
Epidural catheter, *n*		79 (21%)	60 (10%)
Hospital length of stay, days		6 (3, 10)	5 (3, 9)
Advanced post anaesthesia care unit stay, *n*		123 (33%)	148 (24%)

Data are shown as absolute number (percentage) or median (25^th^, 75^th^ percentile).

### Primary outcome: postoperative hypotension

**Fig 2:**
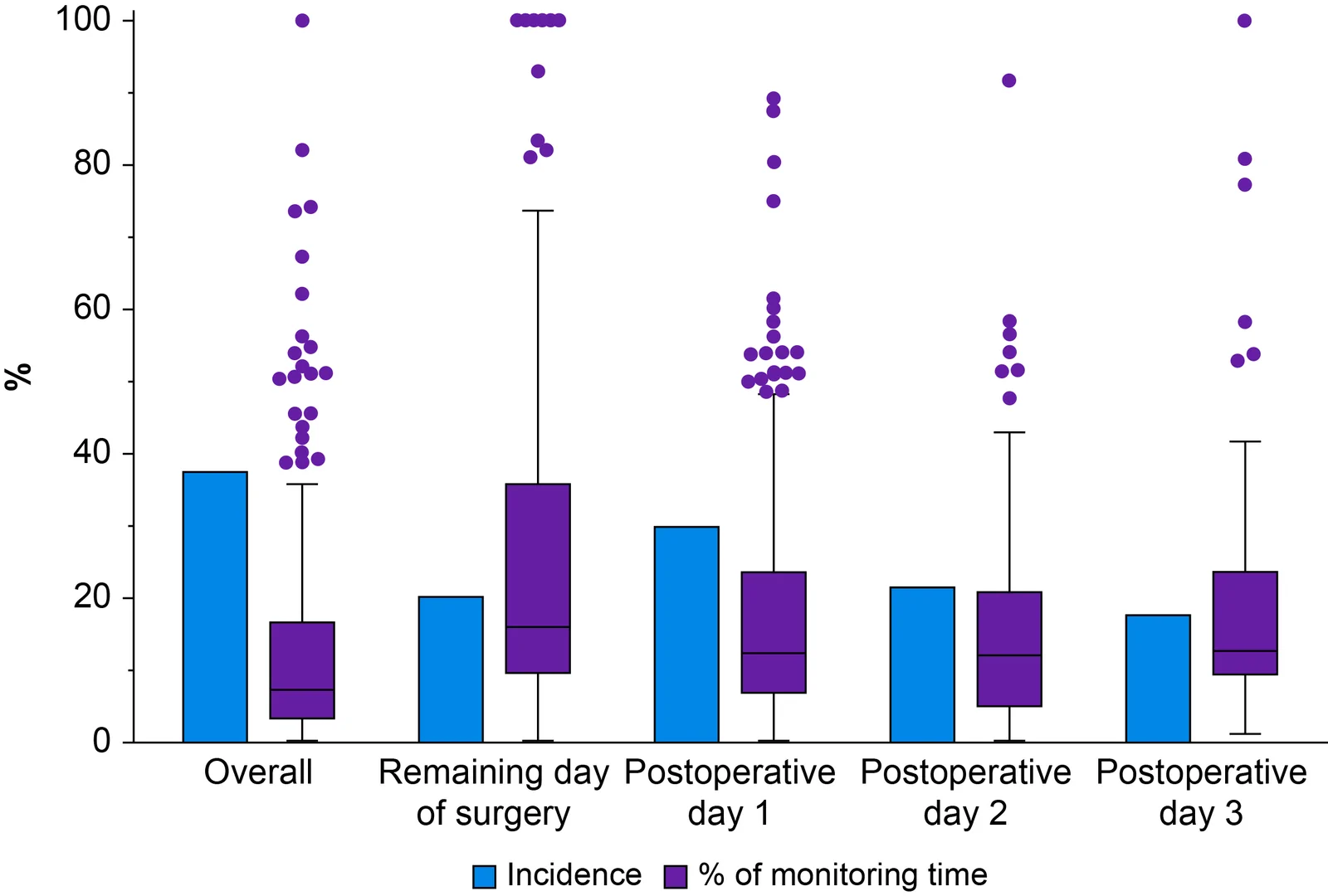
Postoperative hypotension with mean arterial pressure <65 mm Hg. Bar chart illustrating the incidence and box plots illustrating the percentage of monitoring time with mean arterial pressure <65 mm Hg for the remaining day of surgery (at risk *n*=1000); postoperative day 1 (at risk *n*=913); postoperative day 2 (at risk *n*=657); postoperative day 3 (at risk *n*=380). The percentage of monitoring time with a mean arterial pressure <65 mm Hg is shown as median with lower and upper box limit indicating the 25^th^ and 75^th^ percentile only for participants with at least one mean arterial pressure <65 mm Hg.

A total of 378 participants (37.7%) had postoperative hypotension defined as any MAP <65 mm Hg within the first 3 postoperative days. The median cumulative duration for participants with postoperative hypotension was 180 (71, 420) min, corresponding to 7.1% (3.3%, 16.3%) of their monitoring time. The median area under a MAP of 65 mm Hg was 836 (300, 2037) mm Hg x min. The median time-weighted average MAP <65 mm Hg was 0.32 (0.12, 0.73) mm Hg. The incidence of postoperative hypotension progressively decreased from postoperative day 1 to day 3, whereas its duration and severity did not (Fig. 2, Table 2; Supplementary Fig. 1). Within the first 3 postoperative days, 734 participants (73.3%) had at least one MAP <75 mm Hg and 91 participants (9.1%) had at least one MAP <55 mm Hg (Table 3, Supplementary Table 1).

**Table 2:** Summary of postoperative hypotension. Data are shown as absolute number (percentage) or median (25th, 75th percentile). MAP, mean arterial pressure; TWA, time weighted average. ∗Only participants with MAP measurement values below threshold included.

	Overall (*n*=1002)	Remaining day of surgery (*n*=1000)	Postoperative day 1 (*n*=913)	Postoperative day 2 (*n*=657)	Postoperative day 3 (*n*=380)
Incidence of MAP <65 mm Hg, *n*	378 (37.7%)	202 (20.2%)	273 (30.0%)	140 (21.3%)	65 (17.1%)
Duration with MAP <65 mm Hg∗, min	180 (71, 420)	100 (60, 180)	160 (62, 300)	120 (60, 183)	62 (60, 123)
Percentage of monitoring time with MAP <65 mm Hg∗, %	7.1 (3.3, 16.3)	15.9 (9.6, 35.2)	12.5 (7.1, 23.5)	12.0 (5.2, 20.8)	12.8 (9.6, 23.7)
Area under a MAP of 65 mm Hg∗, mm Hg x min	836 (300, 2037)	366 (177, 933)	568 (238, 1380)	575 (193, 1148)	348 (120, 766)
TWA MAP <65 mm Hg∗, mm Hg	0.32 (0.12, 0.73)	0.67 (0.28, 1.54)	0.46 (0.17, 1.05)	0.52 (0.24, 1.03)	0.51 (0.21, 1.12)

**Table 3:** Summary of postoperative mean arterial pressure <55 mm Hg and <75 mm Hg. Data are shown as median (25th, 75th percentile) or absolute number (percentage). MAP, mean arterial pressure; TWA, time weighted average. ∗only participants with MAP values below threshold included.

	<55 mm Hg	<75 mm Hg
Incidence of MAP below threshold, *n*	91 (9.1%)	734 (73.3%)
Duration with MAP below threshold∗, min	60 (60, 120)	480 (182, 1052)
Percentage of monitoring time with MAP below threshold∗, %	2.5 (1.6, 4.9)	20.1 (8.3, 41.3)
Area under MAP below threshold∗, mm Hg x min	300 (120, 509)	2548 (840, 6903)
TWA MAP below threshold∗, mm Hg	0.09 (0.04, 0.17)	1.05 (0.32, 2.68)

### Exploratory outcomes

Postoperative complications within 7 days after surgery, according to the Clavien-Dindo classification, occurred in 107 participants (28.3%) with postoperative hypotension, compared with 169 participants (27.1%) without postoperative hypotension (*P*=0.728). Postoperative acute kidney injury (assessed in 852 participants) occurred in 34 participants (10.5%) with postoperative hypotension, compared with 39 participants (7.4%) without postoperative hypotension (*P*=0.154). Unplanned admission to the intensive care unit was necessary in 15 participants (4.0%) with postoperative hypotension, compared with eight participants (1.3%) without postoperative hypotension (*P*=0.011). Mortality within 30 days (assessed in 852 participants) was four participants (1.3%) with and three participants (0.6%) without postoperative hypotension (*P*=0.528). The median hospital length of stay was 6 (3, 10) days in participants with and 5 (3, 9) days in participants without postoperative hypotension (*P*=0.057).

## Discussion

In this prospective observational study of adults recovering from noncardiac surgery, hypotension (defined as MAP <65 mm Hg) within the first 3 postoperative days was common – but rarely severe or prolonged. The incidence of postoperative hypotension progressively decreased from postoperative day 1 to day 3.

We primarily defined postoperative hypotension as a MAP <65 mm Hg within the first 3 postoperative days. The incidence of postoperative hypotension varies depending on the setting, study population, duration of monitoring and the MAP threshold used to define hypotension.^5^^,^^15^^,^^16^ Nevertheless, the ∼40% incidence of postoperative hypotension we found aligns with previous studies. In a prospective observational study, blinded automated blood pressure monitoring revealed a 38% incidence of postoperative hypotension within the first 2 postoperative days in 312 abdominal surgery patients.^17^ In another prospective study, 41% of 248 patients recovering from noncardiac surgery on general wards had postoperative hypotension within the first 24 postoperative hours on automated oscillometric monitoring at 30-min intervals.^10^ In contrast, a retrospective analysis of 67 968 noncardiac surgeries found a 22% incidence of postoperative hypotension with a MAP <65 mm Hg within 48 h after surgery based on routine spot-check blood pressure measurements.^18^ In this retrospective analysis, postoperative hypotension most frequently occurred within the first 4 h after surgery. This is in contrast to our study, in which the incidence, duration, and severity of postoperative hypotension were highest on postoperative day 1—and then decreased. Importantly, monitoring durations were shorter on the remaining day of surgery, and progressively fewer participants were monitored on the later days. While the incidence and absolute duration decreased, the percentage of monitoring time with a MAP <65 mm Hg and the time-weighted average MAP <65 mm Hg were similar across the 3 postoperative days.

We also quantified postoperative hypotension as the area under a MAP of 65 mm Hg and the time-weighted average MAP <65 mm Hg, which are particularly informative metrics because they integrate both the duration and severity of hypotension. The time-weighted average additionally considers the total monitoring time. However, a given value of the area under a MAP of 65 mm Hg or the time-weighted average MAP <65 mm Hg can result from prolonged periods with moderately low pressures or from short periods with profoundly low pressures. It is, thus, important to interpret these values together with the hypotension duration. The amount of postoperative hypotension we observed vastly exceeds the amounts of intraoperative hypotension in recent large trials.^19^^,^^20^

The optimal MAP threshold to define postoperative hypotension remains unknown. In our study, MAPs <55 mm Hg, reflecting more severe postoperative hypotension, occurred in only about one of 10 participants, and most observed episodes were short. In contrast, MAPs <75 mm Hg were common, and episodes were often prolonged. While these episodes may represent less severe physiological derangement on an absolute scale, their prolonged duration and high cumulative exposure time could potentially contribute to end-organ hypoperfusion and subsequent complications. Conversely, low blood pressures may also reflect normal physiological variations in healthy persons, e.g., during nocturnal dipping. It was previously found that about half of patients with postoperative hypotension also had blood pressures below 65 mm Hg before surgery, which raises the question of whether all postoperative hypotension is harmful and clinically meaningful.^10^ These observations highlight the need to distinguish potentially harmful hypotension from benign, context-dependent blood pressure decreases. Without understanding each patient’s baseline haemodynamic profile and circadian patterns, it remains difficult to interpret postoperative MAP perturbations, potentially overstating the clinical significance of transient reductions that fall within an individual’s normal range.

On an exploratory basis, we assessed postoperative complications. Participants with and without postoperative hypotension had similar rates of postoperative complications according to the Clavien-Dindo classification. However, participants with postoperative hypotension had higher rates of acute kidney injury and unplanned intensive care unit admission than participants without postoperative hypotension. As postoperative hypotension may be a symptom, but could also be a cause of postoperative complications, further research is necessary to investigate whether there is a causal relationship between postoperative hypotension and complications and if strategies to avoid postoperative hypotension can help reduce complications. Even though targeting intraoperative blood pressure higher than 65 mm Hg does not improve postoperative outcomes,^19^^,^^21^^,^^22^ it remains unknown whether targeted postoperative blood pressure management may help reduce organ injury.

We monitored postoperative blood pressure using automated oscillometry at 1-h intervals for the first 3 postoperative days. Oscillometry is considered a reference method for automated blood pressure monitoring.^23^, ^24^, ^25^ We cannot rule out that intermittent oscillometric blood pressure measurements missed some hypotension episodes. Yet, continuous noninvasive blood pressure monitoring on general wards remains challenging^9^ and innovative noninvasive continuous blood pressure monitoring methods do not yet provide reliable measurements.^23^^,^^24^

Our study has several additional limitations. We recorded postoperative complications according to Clavien-Dindo and analysed acute kidney injury based on creatinine levels—but did not systematically investigate other complications that may potentially be associated with or caused by hypotension, such as acute myocardial injury.^11^ Our analysis was not powered to assess the relationship between postoperative hypotension and complications. Even with a larger sample size, the observational design of our study would not allow establishing a causal relationship between postoperative hypotension and complications. Finally, we did not investigate the causes of postoperative hypotension.

In summary, during recovery from noncardiac surgery, we found that postoperative hypotension was common but rarely severe or prolonged. The incidence of postoperative hypotension progressively decreased from postoperative day 1 to day 3. Large-scale trials are needed to evaluate whether continuous postoperative blood pressure monitoring and targeted postoperative blood pressure management can help improve patient-centred outcomes.

## Authors’ contributions

Study conception/design: MF, BS

Data acquisition: MF, SS, MB, BÜ

Data analysis: all authors

Drafting of the manuscript: MF, BS

Critical revision of the manuscript for important intellectual content: all authors.

All authors reviewed the results and approved the final version of the manuscript.

## Declaration of generative AI and AI-assisted technologies in the writing process

During the preparation of this work, the authors used Claude Sonnet 4.5 (Anthropic; San Francisco, CA, USA) to improve readability and language. The authors reviewed and edited the content as needed and take full responsibility for the content of the publication.

## Funding

GE Healthcare (Chicago, IL, USA; institutional restricted research grant).

## Declarations of interest

MF is a consultant for Edwards Lifesciences (Irvine, CA, USA) and Vygon (Écouen, France) and has received honoraria for consulting and giving lectures from CNSystems Medizintechnik (Graz, Austria). KKT has received honoraria for consulting and for giving lectures from Masimo (Neuchâtel, Switzerland). BS is a consultant for Edwards Lifesciences (Irvine, CA, USA), Philips North America (Cambridge, MA, USA), GE Healthcare (Chicago, IL, USA), Vygon (Aachen, Germany), Masimo (Neuchâtel, Switzerland), Retia Medical (Valhalla, NY, USA), Maquet Critical Care (Solna, Sweden), Pulsion Medical Systems (Feldkirchen, Germany), Dynocardia (Cambridge, MA, USA), RDS (Strasbourg, France). BS has received institutional restricted research grants from Edwards Lifesciences, Baxter (Deerfield, IL, USA), GE Healthcare, Masimo, Philips Medizin Systeme Böblingen (Böblingen, Germany), CNSystems Medizintechnik (Graz, Austria), Pulsion Medical Systems, Vygon, Retia Medical, Osypka Medical (Berlin, Germany). BS has received honoraria for giving lectures from Edwards Lifesciences, Philips Medizin Systeme Böblingen, Baxter, GE Healthcare, Masimo, CNSystems Medizintechnik, Getinge (Gothenburg, Sweden), Pulsion Medical Systems, Vygon, Ratiopharm (Ulm, Germany). BS is an Editor of the British Journal of Anaesthesia. The other authors have no conflicts of interest to declare.
