# Supplementary material for: Postoperative hypotension in patients recovering from noncardiac surgery: a prospective, blinded observational study
Source: Br J Anaesth. 2026 Jun 1;137(1):66–73. doi: 10.1016/j.bja.2026.04.042 (PMC13494075; doi:10.1016/j.bja.2026.04.042)
Supplement: Supplementary file 1 [file mmc1.pdf]

Supplementary Figure 1. Postoperative hypotension until postoperative day 3.

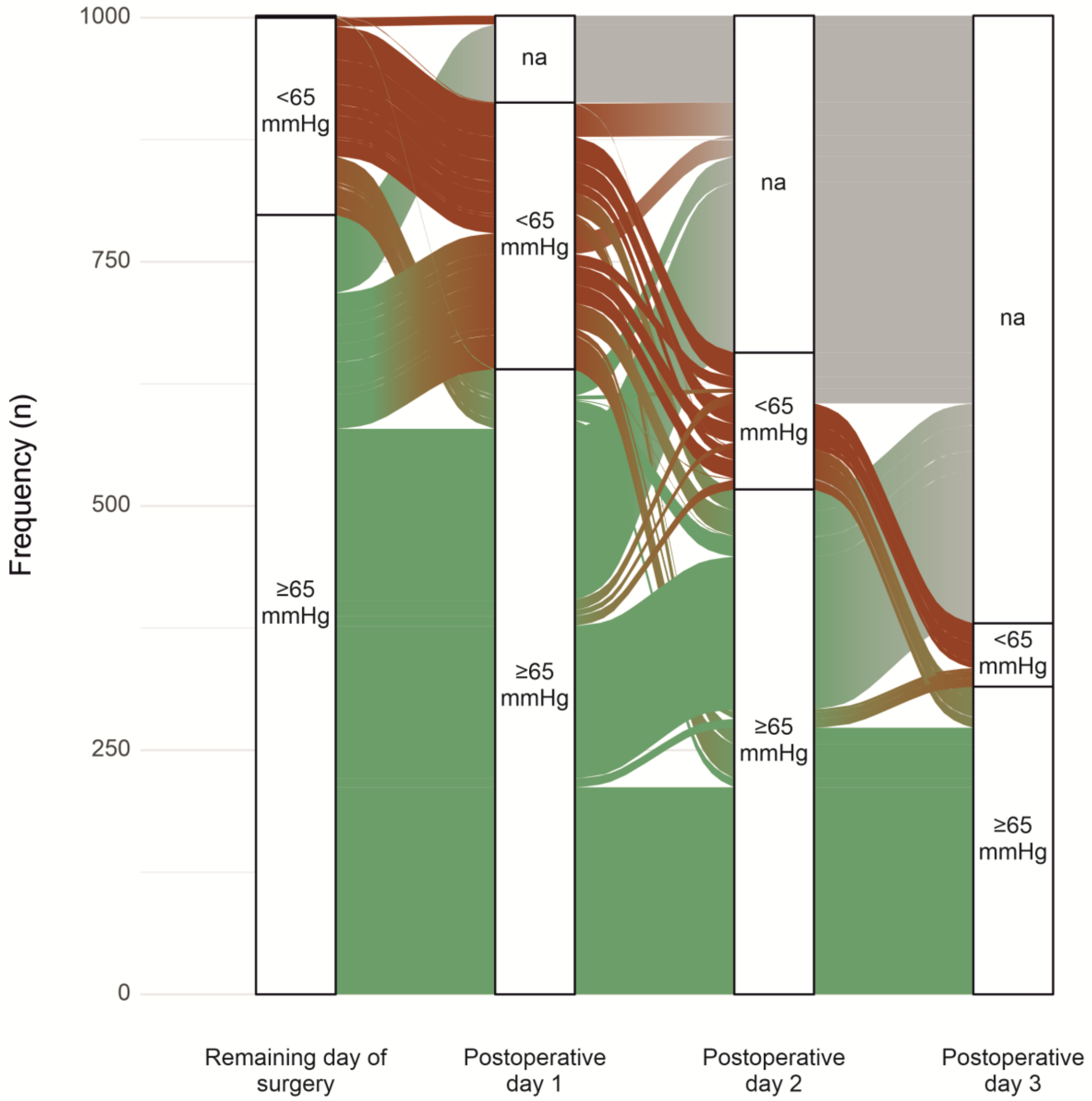

Sankey diagram illustrating patients with (red) and without (green) at least one MAP <65 mmHg from the remaining day of surgery until postoperative day 3. Grey bands indicate patients without blood pressure measurements on that day.
