# Supplementary material for: Postoperative hypotension in patients recovering from noncardiac surgery: a prospective, blinded observational study
Source: Br J Anaesth. 2026 Jun 1;137(1):66–73. doi: 10.1016/j.bja.2026.04.042 (PMC13494075; doi:10.1016/j.bja.2026.04.042)
Supplement: Supplementary file 2 [file mmc2.pdf]

	**Overall** (*n*=1002)	**Remaining day of surgery** (*n*=1000)	**Postoperative day 1** (*n*=913)	**Postoperative day 2** (*n*=657)	**Postoperative day 3** (*n*=380)
55 mmHg					
Incidence of MAP below threshold, n	91 (9.1%)	26 (2.6%)	53 (5.8%)	25 (3.8%)	8 (2.1%)
Duration with MAP below threshold*, min	60 (60, 120)	60 (60, 63)	60 (60, 120)	60 (60, 63)	78 (60, 137)
Percentage of monitoring time with MAP below threshold*, %	2.5 (1.6, 4.9)	8.3 (4.2, 16.0)	4.3 (4.2, 8.5)	4.4 (4.2, 12.1)	13.0 (9.6, 14.1)
Area under MAP*, mmHg x min	300 (120, 509)	180 (61, 285)	300 (124, 480)	240 (120, 360)	303 (179, 540)
TWA MAP*, mmHg	0.09 (0.04, 0.17)	0.24 (0.09, 0.37)	0.21 (0.09, 0.42)	0.20 (0.13, 0.43)	0.38 (0.27, 0.70)
75 mmHg					
Incidence of MAP below threshold, n	734 (73.3%)	515 (51.5%)	612 (67.0%)	384 (58.4%)	187 (49.2%)
Duration with MAP below threshold*, min	480 (182, 1052)	180 (62, 360)	314 (123, 597)	192 (93, 393)	123 (61, 259)
Percentage of monitoring time with MAP below threshold*, %	20.1 (8.3, 41.3)	37.5 (15.8, 68.1)	26.8 (12.3, 50.0)	20.8 (8.3, 41.2)	25.3 (12.4, 44.0)
Area under MAP*, mmHg x min	2548 (840, 6903)	858 (360, 2580)	1591 (540, 4055)	1031 (300, 2530)	720 (182, 1823)
TWA MAP*, mmHg	1.05 (0.32, 2.68)	1.84 (0.70, 4.26)	1.41 (0.44, 3.34)	0.91 (0.31, 2.51)	1.11 (0.36, 3.00)

*only patients with MAP values below threshold

Data are shown as absolute number (percentage) or median (25th, 75th percentile). *MAP – mean arterial pressure; TWA – time weighted average*
